# Adaptive stochastic Gauss–Newton method with optical Monte Carlo for quantitative photoacoustic tomography

**DOI:** 10.1117/1.JBO.27.8.083013

**Published:** 2022-04-08

**Authors:** Niko Hänninen, Aki Pulkkinen, Simon Arridge, Tanja Tarvainen

**Affiliations:** a University of Eastern Finland, Department of Applied Physics, Kuopio, Finland; b University College London, Department of Computer Science, London, United Kingdom

**Keywords:** quantitative photoacoustic tomography, Monte Carlo, inverse problems, stochastic optimization

## Abstract

**Significance:**

The image reconstruction problem in quantitative photoacoustic tomography (QPAT) is an ill-posed inverse problem. Monte Carlo method for light transport can be utilized in solving this image reconstruction problem.

**Aim:**

The aim was to develop an adaptive image reconstruction method where the number of photon packets in Monte Carlo simulation is varied to achieve a sufficient accuracy with reduced computational burden.

**Approach:**

The image reconstruction problem was formulated as a minimization problem. An adaptive stochastic Gauss–Newton (A-SGN) method combined with Monte Carlo method for light transport was developed. In the algorithm, the number of photon packets used on Gauss–Newton (GN) iteration was varied utilizing a so-called norm test.

**Results:**

The approach was evaluated with numerical simulations. With the proposed approach, the number of photon packets needed for solving the inverse problem was significantly smaller than in a conventional approach where the number of photon packets was fixed for each GN iteration.

**Conclusions:**

The A-SGN method with a norm test can be utilized in QPAT to provide accurate and computationally efficient solutions.

## 1 Introduction

Photoacoustic tomography (PAT) is an imaging modality based on the photoacoustic effect.^1^^–^^3^ In PAT, images of an initial pressure distribution are reconstructed from boundary measurements of generated photoacoustic waves caused by absorption of an externally induced light pulse. PAT can be applied, e.g., to image soft biological tissues, such as blood vessels and microvasculature of tumors in medical imaging, and for small animal imaging in biomedical applications.^2^^,^^4^^–^^9^ In quantitative photoacoustic tomography (QPAT), the aim is to estimate the concentration of chromophores from photoacoustic images.^10^ This provides more accurate information of the imaged target, such as differentiation between oxygenated and non-oxygenated blood.

Estimation of the chromophore concentrations is an ill-posed problem that needs to be approached in the framework of inverse problems. The optical inverse problem of QPAT is typically formulated as a minimization problem that is solved using methods of numerical optimization.^10^ The chromophore concentrations can be estimated directly from photoacoustic images obtained at multiple wavelengths, or by first reconstructing the absorption coefficients from the photoacoustic images and then computing the concentrations utilizing the absorption spectra of the known chromophores.^10^^–^^17^ Alternatively, the optical parameters can be estimated directly from the photoacoustic time series.^18^^–^^23^ To obtain accurate reconstructions, light propagation in the imaged target needs to be modeled.^12^^,^^24^

A widely accepted forward model for light propagation in a scattering medium, such as biological tissue is the radiative transfer equation (RTE).^25^ Utilizing the RTE in QPAT has been studied, for example in Refs. 21, 24, 26–28. RTE can be solved analytically in a limited number of cases, but it is usually approached numerically using for example a finite element method. In addition to deterministic methods, Monte Carlo method for light transport can be used to simulate light propagation in tissues. Monte Carlo is a stochastic method where light transport is approximated by tracing paths of a large number of photons or photon packets in the medium.^29^ It has been widely utilized in biomedical optics, see e.g., Refs. 30–33 and the references therein. In addition, there is an increasing interest in its usage in solving the inverse problems related to optical imaging, see e.g., Refs. 34–40.

In this work, we study the utilization of Monte Carlo method in the optical inverse problem of QPAT. The approach has been previously studied in Refs. 37–42 either for estimating absorption only or both absorption and scattering. However, despite the recent work,^40^ the number of photon packets have not been investigated, and thus the computational burden of using Monte Carlo for both the forward and inverse problems has been large.

In Monte Carlo, the computational burden is strongly related to the number of simulated photons. Therefore, by adjusting the amount of simulated photons, the computational cost of a Monte Carlo algorithm can be controlled. However, due to the stochastic nature of Monte Carlo, simulating less photons increases stochastic noise in the solution. This trade-off between computational cost and stochastic noise can be used to optimize the computational cost of the approach: if a certain level of noise in the forward model can be accepted, the amount of simulated photons could be chosen to provide sufficient accuracy without unnecessary computational burden.

In the recent work by Macdonald et al.,^40^ efficient image reconstruction strategies using stochastic forward model were investigated. In that work, the QPAT inverse problem was formulated as a least-squares minimization problem for estimating target absorption coefficient. The inverse problem studied was estimation of absorption coefficient based on observation of absorbed energy density in layered (one-dimensional) and noise-free setting. A stochastic gradient descent method was used to solve the minimization problem, and choosing the number of simulated photon packets was studied. In that work, a norm test approach^43^^,^^44^ was used to determine the required number of photon packets to achieve sufficient accuracy of the gradient of the objective function. While the stochastic gradient descent approach was shown to provide accurate estimates in the presented simulations,^40^ utilizing curvature (second-order) information of the objective function, such as Newton’s minimization direction, could provide significantly faster convergence rate, especially in a high-dimensional optimization problem.^45^^–^^47^

In this work, we approach the QPAT inverse problem in the framework of Bayesian inverse problems.^48^^,^^49^ That is, we formulate the inverse problem using models for data likelihood and prior, and seek to find the distribution of target absorption coefficients by computing a maximum a posteriori (MAP) estimate. Inverse problems methodologies, such as the Bayesian framework, enable image reconstruction also in situations where a problem is ill-posed. In this work, we study only the optical inverse problem of QPAT and assume that the initial pressure distribution has been reconstructed, without studying possible reconstruction artefacts caused by the acoustic solver. Further, it is assumed that the scattering coefficient, anisotropy parameter, and the Grüneisen parameter, that is used to describe photoacoustic efficiency, are known.

We formulate an adaptive stochastic Gauss–Newton (A-SGN) method for the solution of the inverse problem. In the approach, the amount of photon packets used by the Monte Carlo forward model in the algorithm is varied on each iteration. We propose an approach where the number of photon packets is determined by a norm test. In the norm test, variance between approximate and accurate minimization direction is studied to determine the number of photon packets. The methodology automatically adjusts the number of photon packets during iteration until a desired convergence of the minimization problem has been achieved.

The rest of the paper is organized as follows. Modeling light transport in QPAT is described in Sec. 2, and the inverse problem of QPAT is described in Sec. 3. In Sec. 4, the stochastic optimization framework and the A-SGN method are presented. Simulation studies are presented in Sec. 5, and results in Sec. 6. Results are discussed and conclusions are given in Sec. 7.

## 2 Forward Model

The optical forward problem in QPAT is to determine the absorbed optical energy density H within the target when the optical parameters and input light are given. In this work, we use Monte Carlo simulations as a forward model to approximate the solution of the RTE.

Let us consider a domain $\Omega \subset R^{d}$ with a boundary ∂Ω in dimension d=2,3 and let $\hat{s}\in S^{d-1}$ denote a unit vector in the direction of interest. In QPAT imaging situation, light propagation in tissue can be modeled using the (time-independent) RTE

{s^·∇ϕ(r,s^)+(μs(r)+μa(r))ϕ(r,s^)=μs(r)∫Sd−1Θ(s^·s^′)ϕ(r,s^′)ds^′,  r∈Ωϕ(r,s^)={ϕ0(r,s^),r∈ϵ,  s^·n^<00,r∈∂Ω∖ϵ,  s^·n^<0, (1)

where r is the spatial position, $\mu_{a}(r)$ is the optical absorption coefficient, $\mu_{s}(r)$ is the optical scattering coefficient, $\phi (r,\hat{s})$ is the radiance, $\phi_{0}(r,\hat{s})$ is a boundary source, $\hat{n}$ is an outward unit normal, and Θ(s^·s^′) is the scattering phase function.^25^^,^^50^^,^^51^ A commonly applied scattering phase function is the Henyey–Greenstein phase function

Θ(s^·s^′)={12π1−g21+g2−2gs^·s^′,d=214π1−g2(1+g2−2gs^·s^′)3/2,d=3, (2)

where −1<g<1 is the scattering anisotropy parameter.^52^ The boundary condition indicates that no photons travel in an inward direction at the boundary except at source position ε⊂∂Ω.

The photon fluence Φ(r) is obtained from the radiance as

Φ(r)=∫Sd−1ϕ(r,s^)ds^. (3)

As light propagates within the medium, it is absorbed by light-absorbing molecules (chromophores), creating absorbed optical energy density H(r)

$$H(r)=\mu_{a}(r)\Phi (r).$$ (4)

The light absorption generates localized increases in pressure that propagate through the tissue. The time evolution of the resulting photoacoustic waves can be modeled using the equations of linear acoustics.^1^

### 2.1 Monte Carlo Method for Light Transport

In this work, we approximate the solution of the RTE with the Monte Carlo method for light transport. We use the photon packet method^29^ implemented in open-source software ValoMC and the associated MATLAB toolbox.^53^ In the photon packet approach, packets of photons with an initial weight $w_{0}$ are generated at light-source locations of the simulation domain.^29^^,^^31^ Scattering distance, or distance for a photon packet to propagate, is drawn from an exponential probability density distribution function

$$f(l)=\mu_{s}(l)\exp(-\int_{0}^{l}\mu_{s}(l^{\prime })dl^{\prime }),$$ (5)

where l is the distance, and $\mu_{s}(l^{\prime })$ is the scattering parameter from the photon packet’s current location toward its current propagation direction. After photon packet has propagated for a scattering distance, a new scattering event occurs where a new propagation direction and a new scattering distance are drawn. In this work, the scattering direction is drawn from the Henyey–Greenstein phase function Eq. (2). These scattering steps are repeated until the photon packet exits the simulation domain or its weight becomes negligible.

During propagation, the photon packet is continuously absorbed by the medium by probability $\mu_{a}ds$ for differential propagation distance ds. That is, the photon weight is described by the Beer–Lambert’s law

$$w(s)=w_{0}\;\exp(-\int_{0}^{s}\mu_{a}(s^{\prime })ds^{\prime }),$$ (6)

which is expressed by parameter s along the photons trajectory, which is formed by a polygonal chain with vertices defined by sequence of scattering locations, with $\mu_{a}(s^{\prime })$ being the absorption coefficient along the trajectory.

In photon packet-based Monte Carlo, the absorbed optical energy density $H_{j}$ in a discretization element j of the domain is computed as

$$H_{j}=-\frac{1}{A_{j}}\int_{0}^{t}\chi_{j}(s)\frac{dw}{ds}(s)ds,$$ (7)

where $A_{j}$ is the area (d=2) or the volume (d=3) of the element j, the integral is understood as being carried from the position where the photon packet was created (s=0) until the photon packet terminates (s=t), $\chi_{j}$ is a characteristic function having the unit value when the photon packet is in the element j and zero elsewhere, and $-\frac{dw}{ds}(s)$ describes the energy absorbed by the medium during the photon packet propagation.

## 3 Inverse Problem

In this work, we focus to study the optical inverse problem of QPAT. That is, we consider our data to be absorbed optical energy density that is obtained as a solution of the acoustic inverse problem of PAT by reconstructing the initial pressure from photoacoustic time series. Further, it is assumed that the Grüneisen parameter is known.

Let us denote the data vector by $H_{\text{data}}=(h_{1},h_{2},\ldots ,h_{M})\in R^{M}$, where M is the number of data, which in the case of QPAT is the number of illuminations multiplied with the number of discretization points to represent the data. Further, let us denote absorption coefficients as $\mu_{a}=(\mu_{a_{1}},\mu_{a_{2}},\ldots ,\mu_{a_{N}})\in R^{N}$ where N is the number of discretization elements in the parameter grid. The discretized observation model with an additive noise model is

$$H_{\text{data}}=H(\mu_{a})+e,$$ (8)

where $H:R^{N}\mapsto R^{M}$ is the discretized forward model that maps optical parameters to data predictions and $e\in R^{M}$ is additive noise.

In the Bayesian approach to inverse problems, all parameters are modeled as random variables. Using Bayes’ formula and following derivation given for example in Ref. 48, the solution of the inverse problem, i.e., the posterior distribution, can be derived. The unknown absorption $\mu_{a}$ and the noise e are modeled as Gaussian distributed $\mu_{a}∼N(\eta_{\mu_{a}},\Gamma_{\mu_{a}})$ and $e∼N(\eta_{e},\Gamma_{e})$, where $\eta_{\mu_{a}}\in R^{N}$ and $\eta_{e}\in R^{M}$ are the means and $\Gamma_{\mu_{a}}\in R^{N\times N}$ and $\Gamma_{e}\in R^{M\times M}$ are the covariance matrices, respectively. Computing the full posterior distribution is typically computationally too expensive in practical tomographic imaging problems. Therefore, point estimates, such as MAP estimate that is used in this work, are considered. Thus, we estimate absorption coefficients by solving a minimization problem

μ^a=arg minμa{12‖Le(Hdata−H(μa)−ηe)‖2+12‖Lμa(μa−ημa)‖2}=arg minμa{u(μa)}, (9)

where $\Gamma_{\mu_{a}}^{-1}=L_{\mu_{a}}^{T}L_{\mu_{a}}$ and $\Gamma_{e}^{-1}=L_{e}^{T}L_{e}$ are the Cholesky decompositions of the inverse of the covariance matrices and $u(\mu_{a})=\frac{1}{2}{\left\|L_{e}(H_{\text{data}}-H(\mu_{a})-\eta_{e})\right\|}^{2}+\frac{1}{2}{\left\|L_{\mu_{a}}(\mu_{a}-\eta_{\mu_{a}})\right\|}^{2}$.

Solving the minimization problem Eq. (9) is a non-linear optimization problem, which can be achieved using for example a gradient descent or Gauss–Newton (GN) method.^45^ In the GN method,^45^ the estimates are updated as

$$\mu_{a}^{\left(i+1\right)}=\mu_{a}^{\left(i\right)}+\alpha^{\left(i\right)}\delta (\mu_{a}^{\left(i\right)}),$$ (10)

where $\alpha^{\left(i\right)}$ is a step size parameter, and $\delta (\mu_{a}^{\left(i\right)})$ is the GN minimization direction on iteration i and parameter $\mu_{a}^{\left(i\right)}$, which is obtained by solving

$$(J^{T}(\mu_{a}^{\left(i\right)})\Gamma_{e}^{-1}J(\mu_{a}^{\left(i\right)})+\Gamma_{\mu_{a}}^{-1})\delta (\mu_{a}^{\left(i\right)})=J^{T}(\mu_{a}^{\left(i\right)})\Gamma_{e}^{-1}(H_{\text{data}}-H(\mu_{a}^{\left(i\right)})-\eta_{e})-\Gamma_{\mu_{a}}^{-1}(\mu_{a}^{\left(i\right)}-\eta_{\mu_{a}}),$$ (11)

where $H(\mu_{a}^{\left(i\right)})$ is the forward solution and $J(\mu_{a}^{\left(i\right)})$ its Jacobian.

## 4 QPAT Optimization Problem in a Stochastic Setting

As it can be seen, solving the minimization problem Eq. (9) requires solutions to the forward model and its Jacobian. Issues, however, arise when using a stochastic forward model. Nevertheless, the minimization problem Eq. (9) can be approached utilizing methods of stochastic optimization.^40^^,^^47^ In this work, we utilize the stochastic Gauss-Newton (SGN) method.

Let us denote the approximation of the forward model as $H_{P}(\mu_{a})$ and its Jacobian as $J_{P}(\mu_{a})$ evaluated at point $\mu_{a}$ and with a number of photon packets P. They can be expressed as

$$H_{P}(\mu_{a})=H(\mu_{a})+\varepsilon_{H(\mu_{a}),P},$$ (12)

$$J_{P}(\mu_{a})=J(\mu_{a})+\varepsilon_{J(\mu_{a}),P},$$ (13)

where $H(\mu_{a})$ is the “accurate” forward model that refers to the (unavailable) asymptotic limit of Monte Carlo with infinite number of photon packets. Similarly, $J(\mu_{a})$ is the Jacobian of the accurate forward model. Errors of the approximative forward model and its Jacobian are $\varepsilon_{H(\mu_{a}),P}$ and $\varepsilon_{J(\mu_{a}),P}$, respectively. These approximations are assumed to be unbiased, i.e.,

$$E\{H_{P}(\mu_{a})\}=H(\mu_{a}),$$ (14)

$$E\{J_{P}(\mu_{a})\}=J(\mu_{a}),$$ (15)

where E denotes the expected value.

In an idealistic situation, the forward model and its Jacobian would be approximated with a very large number of photon packets leading to approximations with errors that can be regarded infinitesimal. However, this would require a significant amount of computational resources, which would be infeasible in practical applications. On the other hand, if we can accept a certain level of error in the forward solution and its Jacobian, computational cost of evaluating the forward model can be reduced by simulating less photon packets. In this work, we study how to optimally choose the number of photon packets to find a feasible compromise between accuracy and computational cost of the minimization algorithm.

### 4.1 Stochastic Gauss–Newton Method

Let us consider the GN method Eqs. (10) and (11) in a stochastic setting. In the SGN method, estimates are updated as

$$\mu_{a}^{\left(i+1\right)}=\mu_{a}^{\left(i\right)}+\alpha^{\left(i\right)}\delta_{P_{i}}(\mu_{a}^{\left(i\right)}),$$ (16)

where $\delta_{P_{i}}(\mu_{a}^{\left(i\right)})$ is the approximative GN minimization direction computed by solving

$$(J_{P_{i}}^{T}(\mu_{a}^{\left(i\right)})\Gamma_{e}^{-1}J_{P_{i}}(\mu_{a}^{\left(i\right)})+\Gamma_{\mu_{a}}^{-1})\delta_{P_{i}}(\mu_{a}^{\left(i\right)})=J_{P_{i}}^{T}(\mu_{a}^{\left(i\right)})\Gamma_{e}^{-1}(H_{\text{data}}-H_{P_{i}}(\mu_{a}^{\left(i\right)})-\eta_{e})-\Gamma_{\mu_{a}}^{-1}(\mu_{a}^{\left(i\right)}-\eta_{\mu_{a}}),$$ (17)

where $H(\mu_{a}^{\left(i\right)})$ is the approximate forward solution and $J_{P_{i}}(\mu_{a}^{\left(i\right)})$ is its Jacobian, approximated with $P_{i}$ photon packets.

To construct the Jacobian, derivatives of absorbed optical energy density with respect to the optical coefficients need to be evaluated. The derivative for the absorption coefficient can be computed directly from Eq. (7) by differentiation. In the case of piece-wise constant absorption $\mu_{a_{k}}$ and absorbed optical energy density $H_{j}$, the derivative can be expressed as

$$\frac{\partial H_{j}}{\partial \mu_{a_{k}}}=-\frac{1}{A_{j}}\int_{0}^{t}\chi_{j}(s)\frac{d}{ds}\frac{\partial w}{\partial \mu_{a_{k}}}(s)ds,$$ (18)

where

$$\frac{\partial w}{\partial \mu_{a_{k}}}(s)=-L_{k}(s)w(s),$$ (19)

and

$$L_{k}(s)=\int_{0}^{s}\chi_{k}(s^{\prime })ds^{\prime },$$ (20)

describes the distance traveled by the photon packets inside element k. For more details, see Ref. 39.

Computing the GN minimization direction is computationally more demanding compared to first-order optimization methods, such as the gradient descent method, as obtaining the GN iteration direction involves solving a linear system Eq. (17). However, utilizing the second-order (curvature) information can result in a much faster convergence in practice.^45^^–^^47^

### 4.2 Adaptive SGN Algorithm with a Norm Test

During the first steps of the SGN iteration, when the absorption estimates are relatively far from the minimum of the optimization problem, even approximative knowledge of the minimization direction can be used to achieve minimization directions that provide sufficient decrease. When the iterations advance and the estimates approach the minimum, the difference between the forward model and data vector decreases. Consequently, the effect of the stochastic noise in the difference increases. If the stochastic noise starts to dominate, the minimization direction computed based on this difference may not be useful and it is possible that the algorithm starts to jump in the surroundings of the minimum. On the other hand, if the accuracy of the minimization direction (number of photon packets) is increased as iterations proceed, the effect of the stochastic noise could be kept sufficiently low.

In this work, we propose an adaptive approach for choosing the number of photon packets on each iteration. The iteration algorithm starts with a relative low number of photon packets $P_{1}$. In addition, in the beginning of the algorithm, the number of samples in the norm test L and initial absorption parameters $\mu_{a}^{\left(1\right)}$ are set. The accuracy of the minimization direction is assessed using a so-called norm test,^43^^,^^44^ and if needed, the number of photon packets is increased. A similar approach has been recently utilized in Ref. 40 to study the number of photon packets needed in a stochastic gradient method in QPAT.

In the norm-test, the expected value of the squared relative error of the approximative minimization direction is controlled. For the SGN method, it can be expressed as

$$V_{P_{i}}(\mu_{a}^{\left(i\right)}{)}^{2}≔\frac{E\{{\left\|\delta (\mu_{a}^{\left(i\right)})-\delta_{P_{i}}(\mu_{a}^{\left(i\right)})\right\|}^{2}\}}{{\left\|\delta (\mu_{a}^{\left(i\right)})\right\|}^{2}}\leq \gamma^{2},\;\gamma >0,$$ (21)

where $\delta (\mu_{a}^{\left(i\right)})$ is the accurate minimization direction, that is a minimization direction that is computed with such a large number of photon packets that it can be regarded exact within measurement precision. Further, $V_{P_{i}}{\left(\mu_{a}^{\left(i\right)}\right)}^{2}$ describes the expected value of the squared relative error evaluated at point $\mu_{a}^{\left(i\right)}$ with $P_{i}$ photon packets and γ is a threshold parameter defining acceptable relative error in the minimization direction.

In practice, the accurate minimization direction $\delta (\mu_{a}^{\left(i\right)})$ is not available. Therefore, on each iteration, we compute L approximate forward solutions $\left\{H_{P_{i}}^{\left(\ell \right)}(\mu_{a}^{\left(i\right)})\right\}$ and Jacobians $\left\{J_{P_{i}}^{\left(\ell \right)}(\mu_{a}^{\left(i\right)})\right\}$ using $P_{i}$ photon packets for ℓ=1,…,L. An approximation of the accurate forward solution $H(\mu_{a}^{\left(i\right)})$ and Jacobian $J(\mu_{a}^{\left(i\right)})$ can be computed from the means of these samples $\left\{H_{P_{i}}^{\left(\ell \right)}(\mu_{a}^{\left(i\right)})\right\}$ and $\left\{J_{P_{i}}^{\left(\ell \right)}(\mu_{a}^{\left(i\right)})\right\}$, which can be used to compute an approximation of the accurate GN direction $\delta (\mu_{a}^{\left(i\right)})$. Then, the samples $\left\{H_{P_{i}}^{\left(\ell \right)}(\mu_{a}^{\left(i\right)})\right\}$ and $\left\{J_{P_{i}}^{\left(\ell \right)}(\mu_{a}^{\left(i\right)})\right\}$ are utilized to compute the value of $V_{P_{i}}(\mu_{a}^{\left(i\right)})$. If the norm test, Eq. (21), fails and the inequality does not hold, the error in the minimization direction is considered to be too large and the number of photon packets is increased. In this work, we use similar method as presented in Ref. 40 where the number of photon packets is increased by a factor

$$P_{i}\leftarrow \frac{V_{P_{i}}{\left(\mu_{a}^{\left(i\right)}\right)}^{2}}{\gamma^{2}}P_{i}.$$ (22)

The algorithm and implementation of the norm test are presented in Algorithm 1. Choice of the parameters used in the adaptive SGN algorithm and in the norm test in this work is discussed in more detail in Sec. 5.

**Algorithm 1:** Adaptive stochastic Gauss–Newton

Set the initial number of photon packets $P_{1}$, number of samples in the norm test L, initial value $\mu_{a}^{\left(1\right)}$ and i←1;
**Repeat**
Compute a set of approximative solutions $\left\{H_{P_{i}}^{\left(\ell \right)}(\mu_{a}^{\left(i\right)})\right\}$ and Jacobians $\left\{J_{P_{i}}^{\left(\ell \right)}(\mu_{a}^{\left(i\right)})\right\}$, ℓ=1,…,L;
Compute a set of approximate GN directions $\left\{\delta_{P_{i}}^{\left(\ell \right)}(\mu_{a}^{\left(i\right)})\right\}$ from $\left\{H_{P_{i}}^{\left(\ell \right)}(\mu_{a}^{\left(i\right)})\right\}$ and $\left\{J_{P_{i}}^{\left(\ell \right)}(\mu_{a}^{\left(i\right)})\right\}$;
Compute an approximation of the accurate GN direction $\delta (\mu_{a}^{\left(i\right)})$ using means of $\left\{H_{P_{i}}^{\left(\ell \right)}(\mu_{a}^{\left(i\right)})\right\}$ and $\left\{J_{P_{i}}^{\left(\ell \right)}(\mu_{a}^{\left(i\right)})\right\}$;
Compute $V_{P_{i}}(\mu_{a}^{\left(i\right)}{)}^{2}$ from Eq. (21);
**if** $V_{P_{i}}(\mu_{a}^{\left(i\right)}{)}^{2}>\gamma^{2}$ **then**
**if** $\frac{V_{P_{i}}(\mu_{a}^{\left(i\right)}{)}^{2}}{\gamma^{2}}>L$ **then**
Set $P_{i}\leftarrow \frac{V_{P_{i}}(\mu_{a}^{\left(i\right)}{)}^{2}}{\gamma^{2}}P_{i}$;
Compute GN direction $\delta_{P_{i}}(\mu_{a}^{\left(i\right)})$ using $P_{i}$ photon packets;
Set $\xi_{i}\leftarrow \delta_{P_{i}}(\mu_{a}^{\left(i\right)})$;
**Else**
Set $P_{i}\leftarrow \frac{V_{P_{i}}(\mu_{a}^{\left(i\right)}{)}^{2}}{\gamma^{2}}P_{i}$;
Set $\xi_{i}\leftarrow \delta (\mu_{a}^{\left(i\right)})$;
**Else**
Set $\xi_{i}\leftarrow \delta (\mu_{a}^{\left(i\right)})$;
Update estimate $\mu_{a}^{\left(i+1\right)}=\mu_{a}^{\left(i\right)}+\xi_{i}$;
Set $P_{i+1}\leftarrow P_{i}$;
Set i←i+1
**until***a convergence criterion is fulfilled*;

It should be noted that computing the norm test Eq. (21) necessitates evaluation of a set of forward model solutions and its Jacobians using multiple photon packets. These can be utilized in the algorithm when the GN search direction is computed after the norm test. That is, the simulated photon packets utilized in the norm test will not be wasted. It is worthy to note that in an optimization algorithm, the step length $\alpha^{\left(i\right)}$ on each iteration should be determined. Newton’s method is associated with a unit step length.^45^ In this work, we also use a step length $\alpha^{\left(i\right)}=1$ to reduce computational cost. Basically this means that we trust the GN approximation of the Hessian to be a good enough approximation to follow the characteristics of the convergence of the Newton’s method. In practice, this leads to a somewhat slower convergence than with an optimal step length but proves savings for the photon packet usage of the algorithm, and thus saves overall computation time.

## 5 Simulation Studies

Image reconstruction problem of QPAT was studied with numerical simulations in two-dimensional (2D) and three-dimensional (3D) domains. Absorption estimates were computed with the SGN method utilizing the proposed adaptive approach for adjusting the number of photon packets. The results were compared to absorption estimates computed using the SGN method without adjusting the number of photon packets during an iteration. The simulations were performed in MATLAB (R2019b MathWorks Inc., Natick, Massachusetts, United States).

### 5.1 Data Simulation

**Fig. 1:**
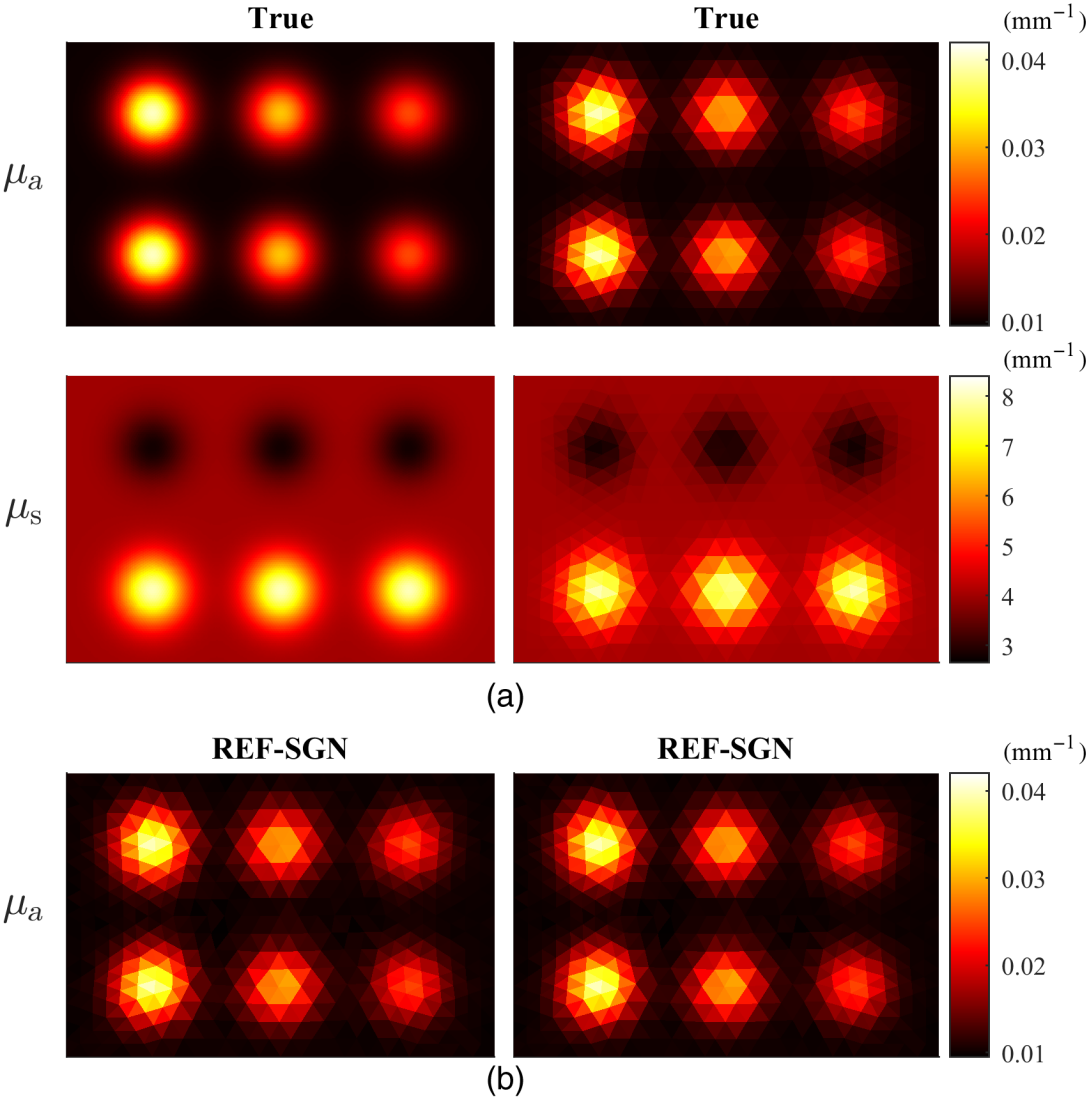
(a) Simulated absorption (first row) and scattering (second row) distributions in the simulation mesh (first column) and in the reconstruction mesh (second column). (b) Reconstructed reference (computed using a large number of photon packets) absorption distribution when the domain was illuminated from all boundaries (left image) and from top and left boundaries (right image).

In the 2D simulations, a rectangular simulation domain of size 15  mm×10  mm was used. The true absorption and scattering distributions, which were used to generate the data, are given in Fig. 1. The scattering anisotropy parameter was g=0.8. To simulate the data, two imaging situations with different illuminations were studied. In the first imaging situation, four planar illuminations, one at each side of the domain, with a uniform intensity covering the whole side length were used. In the second situation, two planar illuminations, at adjacent sides of the domain, with a uniform intensity covering the whole side length were used. The two illumination situations were chosen to simulate the reconstruction problem with different levels of difficulty: one idealistic situation with as many illuminations as possible and the other with an increased ill-posedness due to the limited illumination angle. The absorbed optical energy density was simulated using Monte Carlo method as described in Sec. 2 in a piecewise constant triangular discretization composed of 46,142 elements and 23,360 grid nodes with $10^{9}$ photons packets per illumination. The light source was spatially planar and angularly cosine shape, which means that the initial directions of the photon packets were sampled from a cosine distribution supporting inward directed photon packets. To avoid making an inverse crime, the simulated data were interpolated to a different piecewise constant triangulation that was used as the reconstruction mesh in the inverse problem.

In the 3D simulations, a rectangular domain of size and 15  mm×10  mm×5  mm was used. The true absorption and scattering distributions are shown later in Fig. 12. The scattering anisotropy parameter was g=0.8. Six planar illuminations, one at each side of the domain, were used to simulate the data. The absorbed energy density was simulated using Monte Carlo method similarly as in the 2D simulations, in a discretization composed of 26,244 tetrahedron elements and 5320 nodes and a light source with an angular cosinic shape $10^{9}$ photon packets per illumination were used. The simulated absorbed optical energy density data was interpolated to a reconstruction discretization.

In all simulations, Gaussian radom noise with zero mean and standard deviation corresponding to 1% of the maximum value of noiseless data was added to the simulated data following the interpolation.

### 5.2 Inverse Problem

The inverse problem was solved in the reconstruction mesh. The number of elements and nodes in the different 2D reconstruction discretizations are given in Table 1. In 3D, a mesh composed of 10,920 tetrahedron elements and 2352 nodes was used.

**Table 1:** The number of nodes $N_{n}$ and elements $N_{e}$ of the discretizations $D_{i}$ used in the 2D image reconstruction problem.

	$D_{1}$	$D_{2}$	$D_{3}$	$D_{4}$	$D_{5}$	$D_{6}$
$N_{n}$	260	453	532	1014	1536	2035
$N_{e}$	456	832	972	1900	2914	3888

Following the methodology described in Sec. 3, absorption distributions were reconstructed by minimizing Eq. (9). Two approaches using the SGN method were used: an SGN method where the number of photon packets on each iteration was chosen using the norm test (A-SGN method) and an SGN method with a fixed number of photon packets on each iteration (simple stochastic Gauss–Newton method, S-SGN). To compare the 2D A-SGN and S-SGN estimates to an accurate estimate, a reference absorption estimate was computed by minimizing Eq. (9) using the S-SGN method with an (unnecessary) large number of $10^{8}$ photon packets per iteration. The algorithm for the reference estimate was run for 10 iterations, which ensured its convergence.

The SGN approaches were evaluated in 2D using three different studies. In the first study, the total usage of photon packets by the algorithms, hereinafter referred as a photon budget $P_{b}$, were compared when the algorithms were iterated until converged. In the second study, the performance of the algorithms with equal photon budgets were compared. In both studies, discretization $D_{2}$ was used as a reconstruction mesh. In the third study, computation times of the approaches were studied in different discretizations $D_{i},(i=1,3,\ldots ,6)$ when the algorithms were iterated until converged.

In the A-SGN algorithm, the initial number of photon packets $P_{1}$ was chosen to be 10. The norm test was computed on every iteration to determine the number of photon packets using L=10 samples. In the simulations, where computation times were compared, reconstructions were also computed with the A-SGN approach using only L=5 samples. Further, a threshold parameter γ=0.6 was used. These parameters were chosen based on our observation that they provided accurate reconstructions in the studied simulations. A detailed implementation of the A-SGN was shown in Algorithm 1 in Sec. 3. The number of photon packets in the S-SGN algorithm is presented in Sec. 6.

In addition to the 2D simulations, the feasibility of the approach was validated with a 3D study. The A-SGN and S-SGN methods were used used to reconstruct the absorption distributions by minimising Eq. (9). In the A-SGN algorithm, the same number of samples (L=10) and threshold parameter (γ=0.6) as in the 2D simulations were used, but the initial number of photon packets $P_{1}$ was chosen to be 1000.

In this work, the prior model for absorption was chosen to be based on the Ornstein-Uhlenbeck process.^11^^,^^54^ The Ornstein–Uhlenbeck prior is a Gaussian distribution with the covariance matrix defined as

$$\Gamma_{\mu_{a}}=\sigma_{\mu_{a}}^{2}\Xi ,$$ (23)

where $\sigma_{\mu_{a}}$ is the standard deviation of the prior distribution and Ξ is defined by its elements

$$\Xi (i,j)=\exp(-\|r_{i}-r_{j}\|/\tau ),$$ (24)

where i and j denote the row and column indices of the matrix, respectively, $r_{i}$ and $r_{j}$ denote the element coordinates, and τ is the characteristic length scale parameter. In the reconstructions, the absorption values of the target were assumed to be within an interval $\left[\min(\mu_{a}^{\text{sim}}),\max(\mu_{a}^{\text{sim}})\right]$. The mean of the prior distribution $\eta_{\mu_{a}}$ was chosen to be the mean of that interval, and the standard deviation was chosen such that $\sigma_{\mu_{a}}=1/6(\max(\mu_{a}^{\text{sim}})-\min(\mu_{a}^{\text{sim}}))$. In other words, the interval $\left[\min(\mu_{a}^{\text{sim}}),\max(\mu_{a}^{\text{sim}})\right]$ corresponds to 99.7% of the probability mass of the prior distribution. Characteristic length scale, that controls the spatial smoothness, τ=2.5  mm was used in all simulations. In all simulations, the mean of the prior was also used as the initial guess for the absorption estimates $\mu_{a}^{\left(1\right)}$.

The scattering distribution and the anisotropy parameter were assumed to be known in all simulations, and thus the simulated scattering was interpolated to the reconstruction mesh. Furthermore, the additive noise was assumed well characterized and the estimates were computed with the noise being modeled as zero mean using the simulated noise level.

Since the image reconstruction methodology studied in this work is a stochastic process, the reconstructions were repeated 100 times for the first and second 2D study and five times for the third 2D study to provide statistical information of the approaches. The performance of the algorithms and reconstructed absorption distributions were compared visually and quantitatively. The relative errors of the estimated parameters were computed as

E=100%·‖μ^a−μasim‖‖μasim‖, (25)

where μ^a is the MAP estimate interpolated to the simulation discretization and $\mu_{a}^{\text{sim}}$ is the simulated (true) distribution, and the norm is the Euclidean norm. Further, statistics of the repeated experiments were computed.

## 6 Results

The 2D reference absorption distributions reconstructed using the SGN method with a very large number of photon packets are shown in Fig. 1. These can be regarded as the best possible reconstruction that can be obtained with the current simulation setup.

### 6.1 Comparison of the Photon Budgets When the Convergence Criterion Is Set

In the first study, the photon budget utilized in the A-SGN and S-SGN algorithms was compared. In the A-SGN algorithm, the number of photon packets was adaptively varied as described in Sec. 5. In the S-SGN algorithm, the number of photon packets on each iteration was chosen to be the average number of photon packets in the last A-SGN iteration, which was $6\times 10^{6}$. This was done to ensure the convergence of the S-SGN algorithm to same accuracy as the A-SGN algorithm. It is worthy to notice that in practice this accurate information of the optimal number of photon packets in S-SGN algorithm would not be available. The algorithms were considered converged when the relative difference between the last and three previous absorption estimates was smaller than 10% for all of the three previous estimates. We compared the photon budgets that were required to achieve the convergence criteria.

**Fig. 2:**
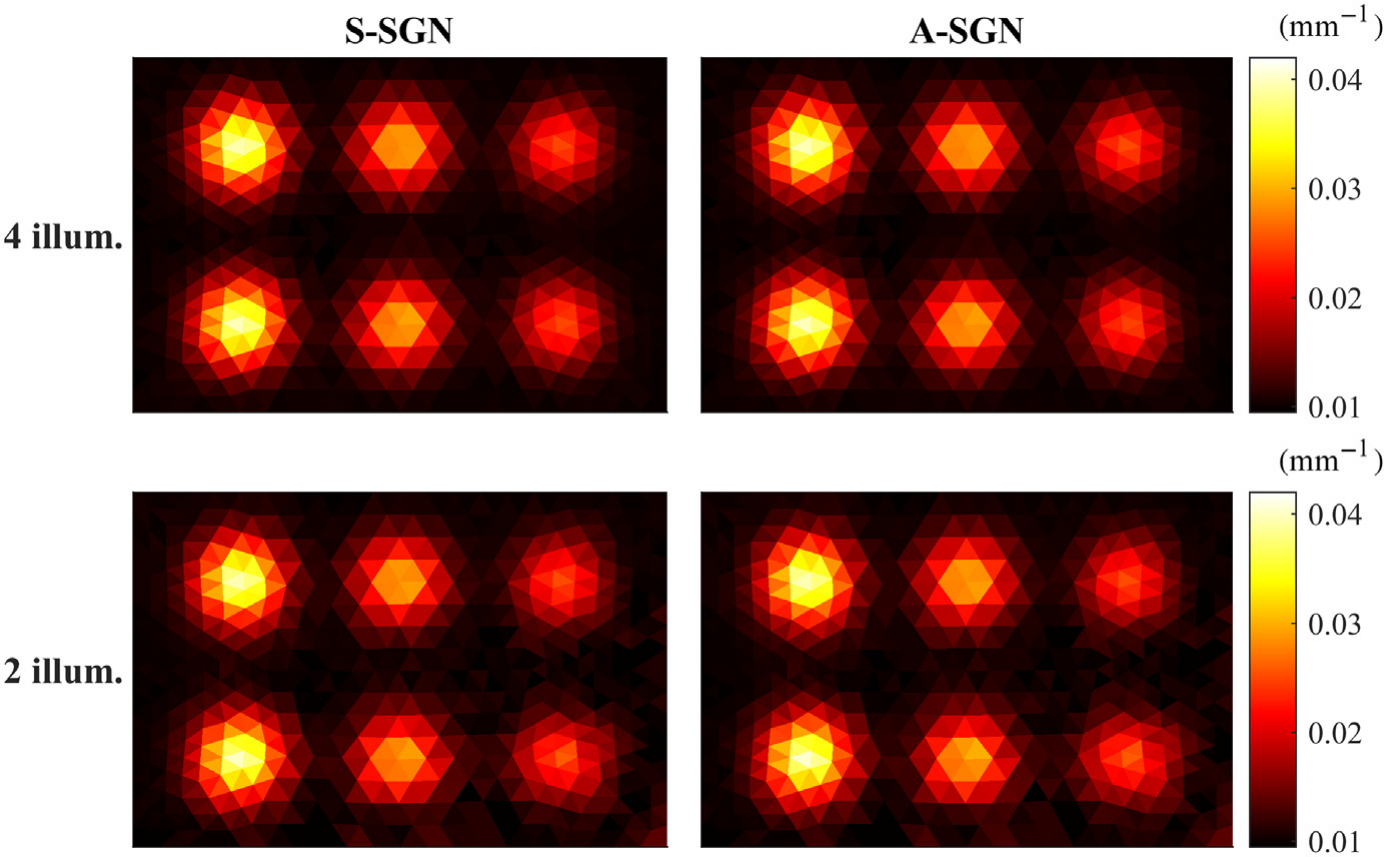
Absorption distribution reconstructed using S-SGN (first column) and A-SGN methods (second column). First row: the domain was illuminated from all boundaries. Second row: the domain was illuminated from top and left boundaries.

The reconstructed absorption coefficients obtained with A-SGN and S-SGN methods are shown in Fig. 2. Both reconstructions look qualitatively identical by visual comparison, which is expected as both algorithms were terminated with the same convergence criterion. Furthermore, they are of the same quality as the reference reconstruction shown in Fig. 1. The reconstructions obtained with four illuminations are slightly better quality than those obtained with two illuminations due to a less ill-posed imaging situation.

**Fig. 3:**
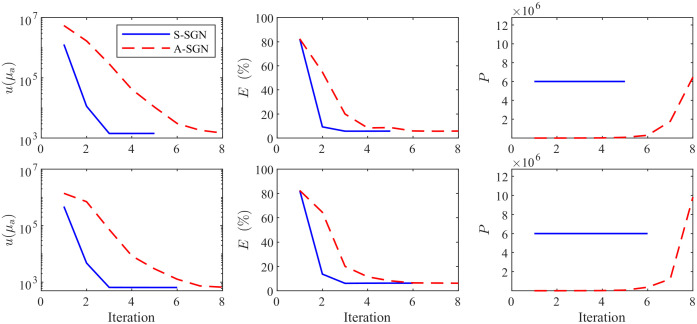
Value of the minimized objective function $u(\mu_{a})$ (first column), relative errors of the estimates E(%) (second column), and the number of photon packets P (third column) as a function of iteration evaluated with the S-SGN (blue solid line) and the A-SGN (red dashed line) methods. First row: the domain was illuminated from all boundaries, second row: the domain was illuminated from top and left boundaries.

The value of the objective function, relative errors of the estimates and number of photon packets as a function of iterations obtained with A-SGN and S-SGN methods are presented in Fig. 3. These results correspond to the simulations shown in Fig. 2. It can been seen that the S-SGN converges with fewer iterations due to the higher number of photon packets per iteration. On the other hand, A-SGN approach requires more iterations to achieve the desired convergence, but the photon budget is significantly smaller. The photon budget used in the A-SGN algorithm was approximately $8.6\times 10^{6}$ in the simulation where the domain was illuminated from all boundaries and $1.2\times 10^{7}$ when the domain was illuminated from two boundaries. In the S-SGN algorithm, the photon budget was $3\times 10^{7}$ in the simulation where the domain was illuminated from all boundaries and $3.6\times 10^{7}$ when the domain was illuminated from two boundaries. Thus, the A-SGN method was able to provide similar reconstructions with a significantly lower photon budget than S-SGN method. It should be noted that even though the initial estimates are identical in both A-SGN and S-SGN approaches, the values of the objective function on the first iteration can be different. This is due to the stochastic nature of the forward model, which affects evaluation of the objective function.

**Fig. 4:**
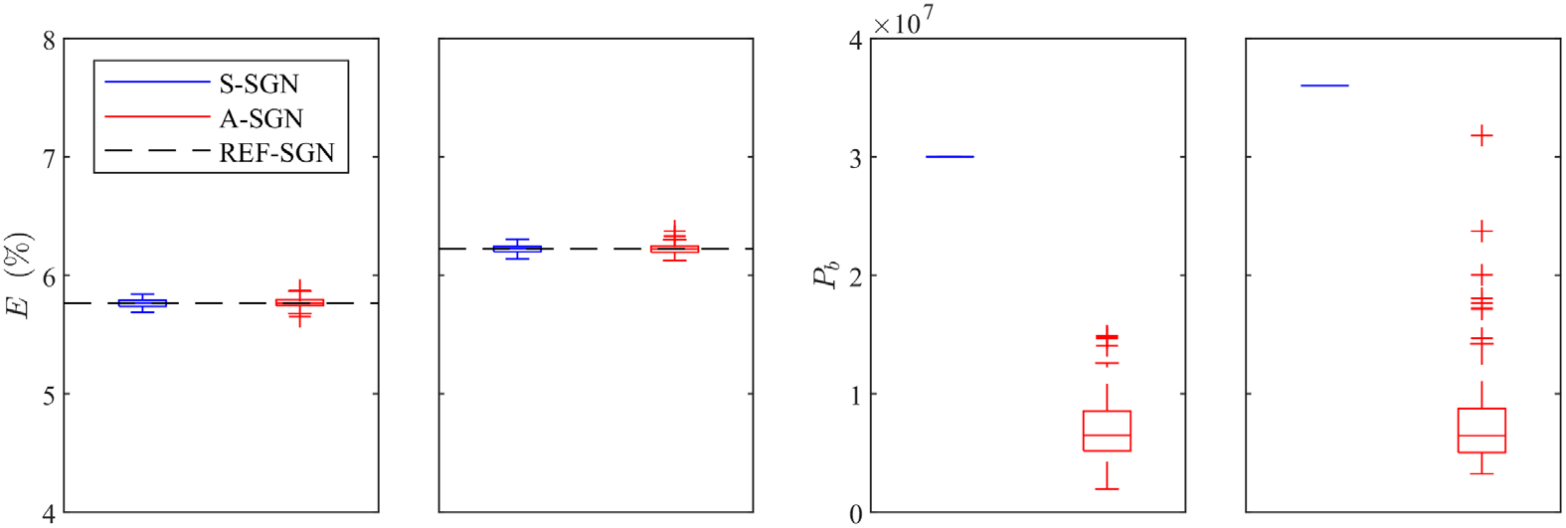
Statistics of the relative errors of the estimates E(%) when the domain was illuminated from all boundaries (first column) and from top and left boundaries (second column) and the statistics of the number of photon packets utilized in the image reconstruction when the domain was illuminated from all boundaries (third column) and from top and left boundaries (fourth column) for 100 evaluation cases. S-SGN method (blue, left) and A-SGN method (red, right), and the reference reconstruction (black horizontal dashed line). The blue and red vertical lines (whiskers) denotes all the samples excluding outliers, box denotes the 25th and 75th percentile, horizontal line denotes the median, and + symbol denotes outliers.

The results shown in Figs. 2 and 3 correspond to one (random) choice from the set of 100 A-SGN and S-SGN reconstructions. The statistics of the relative errors of the reconstructions and photon budgets for the 100 evaluation cases are shown in Fig. 4. As it can be seen, the relative errors are nearly identical in all reconstructions. On the other hand, the A-SGN method is able to achieve these estimates with significantly lower photon budgets in all reconstructions when compared to the S-SGN method.

### 6.2 Comparison of the Reconstruction Accuracy When the Photon Budget Is Set

In the second study, the total number of photon packets utilized in the reconstruction algorithm (the photon budget $P_{b}$), was fixed and equal for both A-SGN and S-SGN methods. In the A-SGN approach, the number of photon packets was determined by the norm test as described in Sec. 5, and the algorithm stopped when the photon budget was used. In the S-SGN approach, the number of simulated photon packets was divided equally for 10 iterations, that is for each iteration the number of photon packets was $P_{i}=P_{b}/10$. In the end of the A-SGN algorithm, if the number of photon packets available in the photon budget was less than required to evaluate the norm test or compute sufficiently accurate direction (as determined by the norm test), the remaining budget was added to the last iteration of the GN algorithm.

**Fig. 5:**
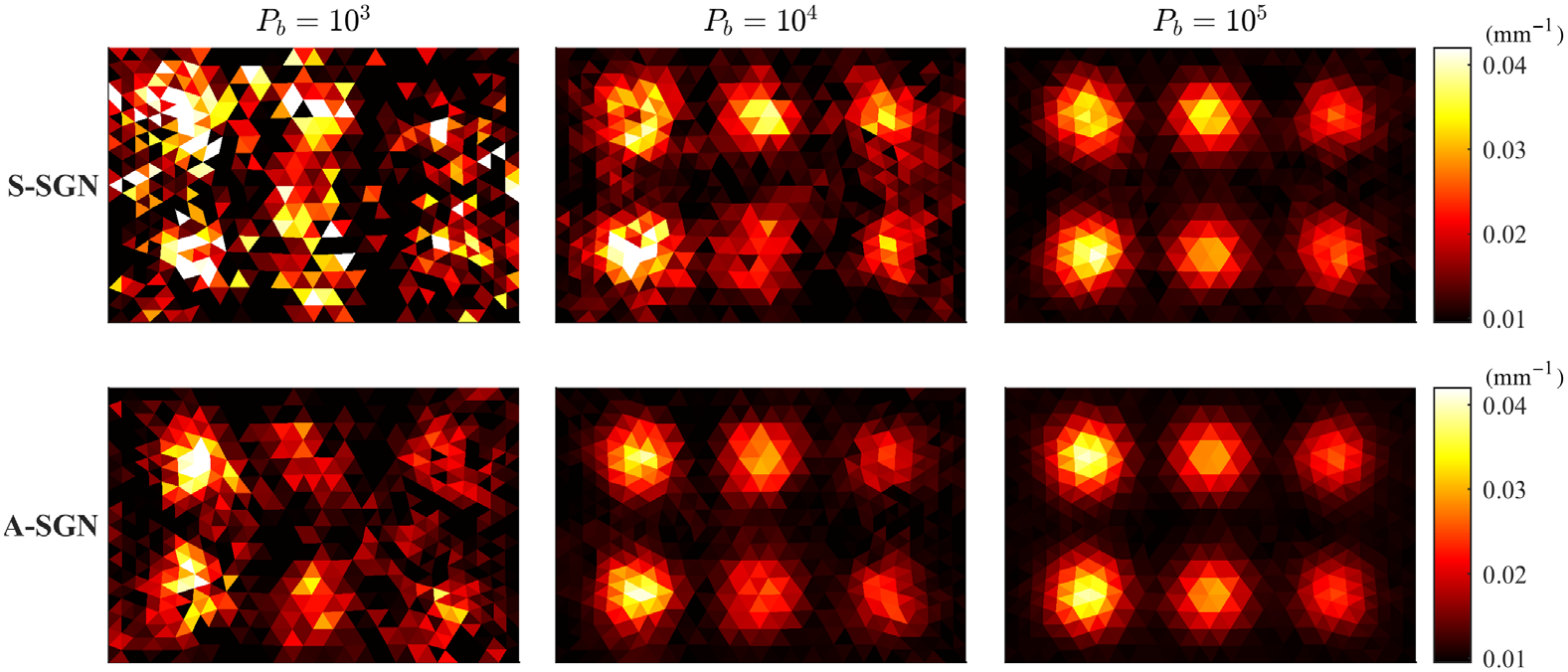
Absorption distributions reconstructed using S-SGN (first row) and A-SGN (second row) methods. In the columns from left to right: the photon budget was $10^{3}$ (first column), $10^{4}$ (second column), and $10^{5}$ (third column). The domain was illuminated from all boundaries.

**Fig. 6:**
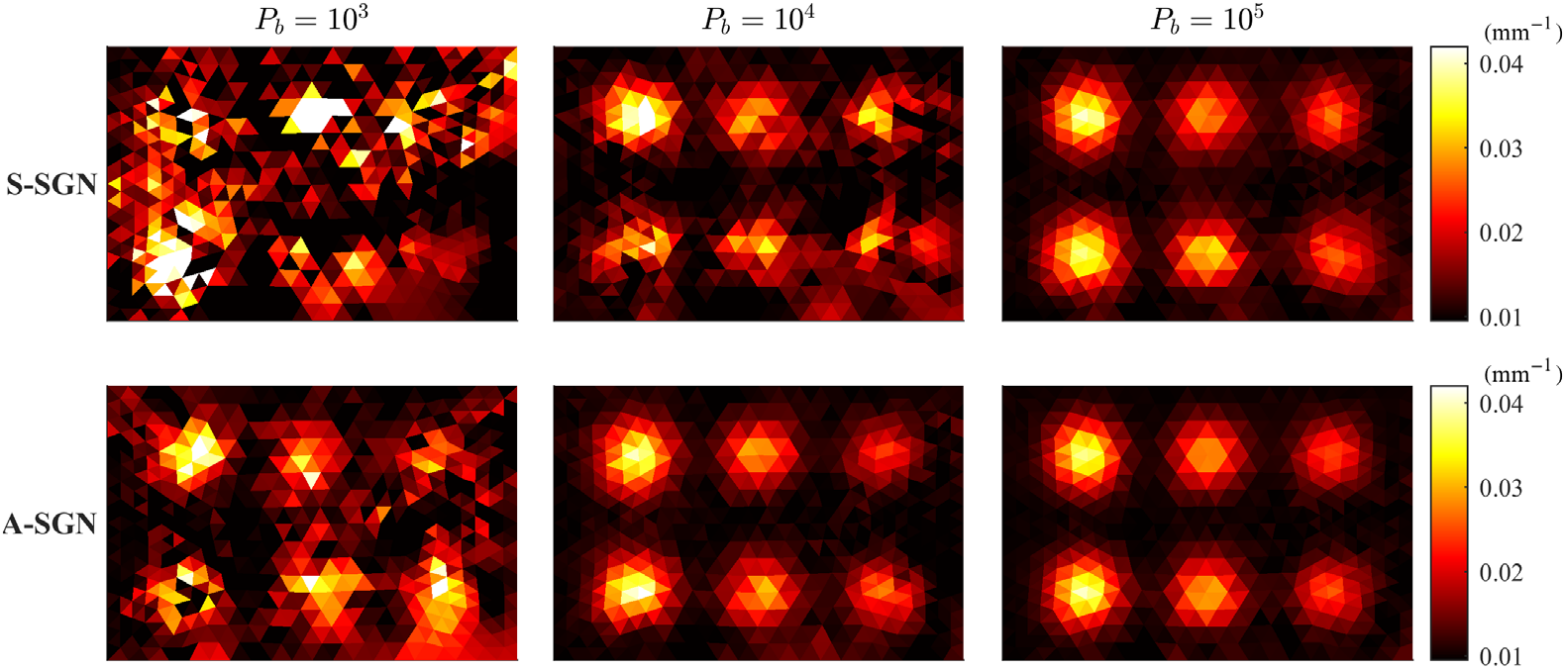
Absorption distributions reconstructed using S-SGN (first row) and A-SGN (second row) methods. In the columns from left to right: the photon budget was $10^{3}$ (first column), $10^{4}$ (second column), and $10^{5}$ (third column). The domain was illuminated from top and left boundaries.

The reconstructed absorption distributions obtained with A-SGN and S-SGN methods with different photon budgets when the domain was illuminated from all boundaries are shown in Fig. 5. Further, the reconstructed absorption distributions when the domain was illuminated from top and left boundaries are shown in Fig. 6. By visual comparison, A-SGN reconstructions are qualitatively more accurate, and this is especially evident with smaller photon budgets. With larger photon budgets, difference between the estimates decreases, and with photon budget of $10^{5}$, reconstructions resemble each other and the reference estimate shown in Fig. 1. The reconstructions obtained with four illuminations are generally slightly better than those obtained with two illuminations.

**Fig. 7:**
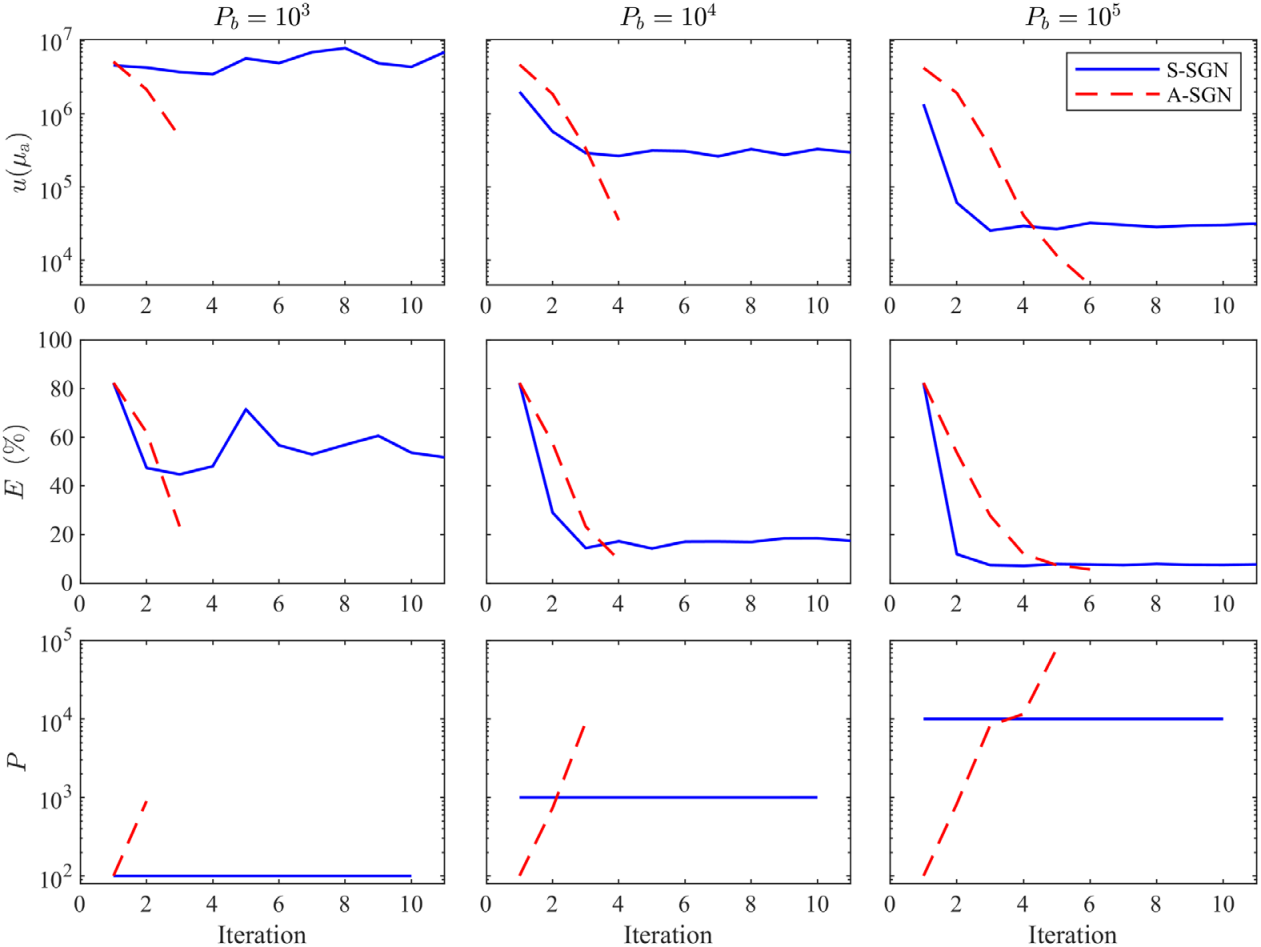
Value of the minimized objective function $u(\mu_{a})$ (first row), relative errors of the estimates E(%) (second row), and the number of photon packets P (third row) as a function of iteration evaluated with the S-SGN (blue solid line) and A-SGN (red dashed line) methods. In the columns from left to right: the photon budget was $10^{3}$ (first column), $10^{4}$ (second column), and $10^{5}$ (third column). The domain was illuminated from all boundaries.

**Fig. 8:**
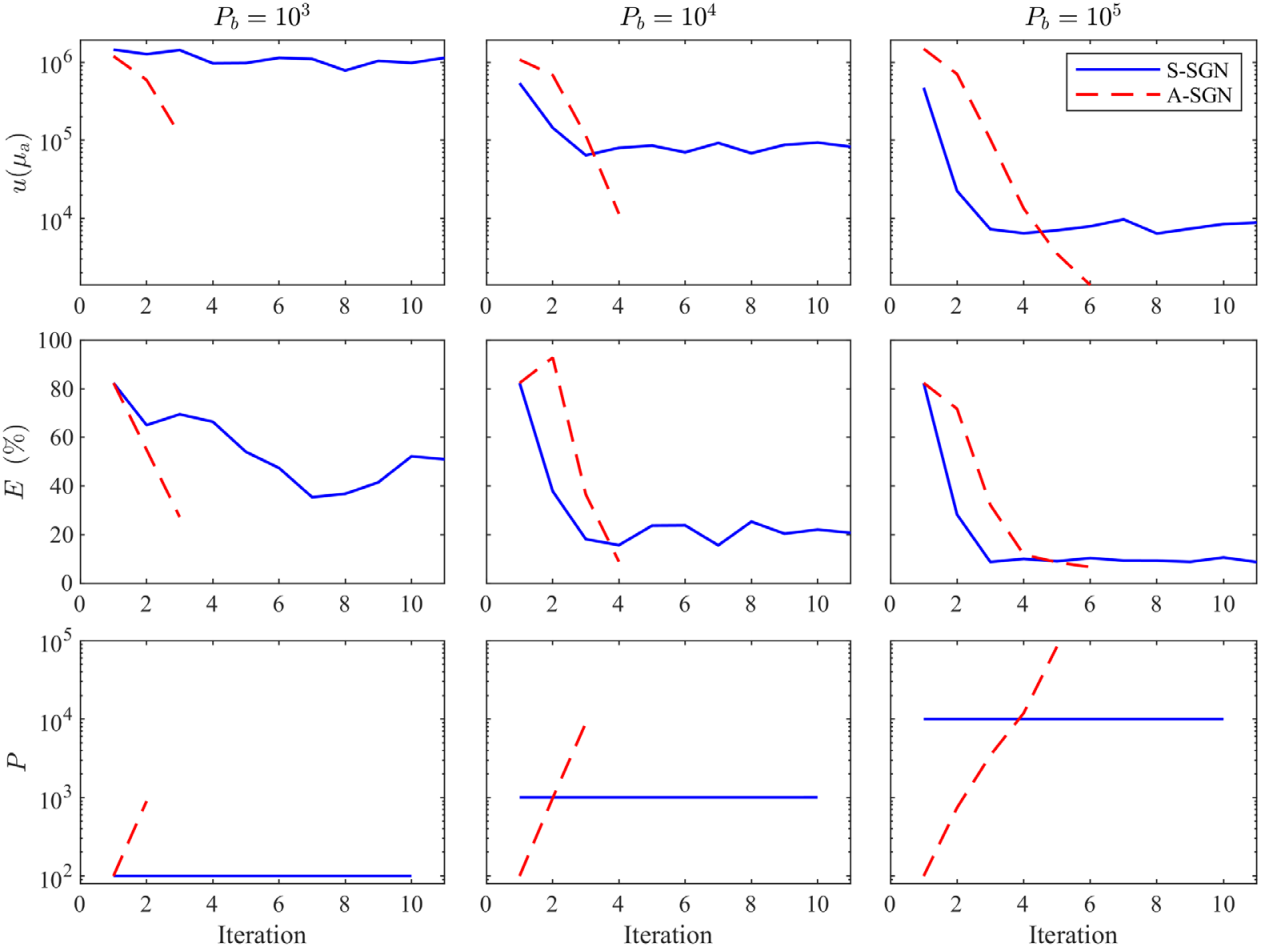
Value of the minimized objective function $u(\mu_{a})$ (first row), relative errors of the estimates E(%) (second row), and the number of photon packets P (third row) as a function of iteration evaluated with the S-SGN (blue solid line) and A-SGN (red dashed line) methods. In the columns from left to right: the photon budget was $10^{3}$ (first column), $10^{4}$ (second column), and $10^{5}$ (third column). The domain was illuminated from top and left boundaries.

The value of the minimized objective function, relative errors of the estimates and the number of photon packets on each iteration corresponding to the reconstruction shown in Fig. 5 where the domain was illuminated from all boundaries are shown in Fig. 7. Further, the value of the minimized objective function, relative errors of the estimates and the number of photon packets on each iteration corresponding to the reconstruction shown in Fig. 6 where the domain was illuminated from top and left boundaries are shown in Fig. 8. As it can be seen in both images, with $10^{3}$ photon budget, the S-SGN is unable to minimize the function effectively. With larger photon budgets, the S-SGN approach minimizes the objective function more effectively and relative errors are smaller during the first iterations due to the larger number of photon packets compared to the A-SGN method. However, after few iterations, the S-SGN approach is unable to achieve more accurate solutions. On the other hand, the A-SGN approach is able to minimize the objective function at every step due to the increasing number of photon packets during the algorithm, and thus is able to achieve more accurate reconstructions. There are no significant differences in the performance of the A-SGN and S-SGN algorithms depending on the number of illuminations.

**Fig. 9:**
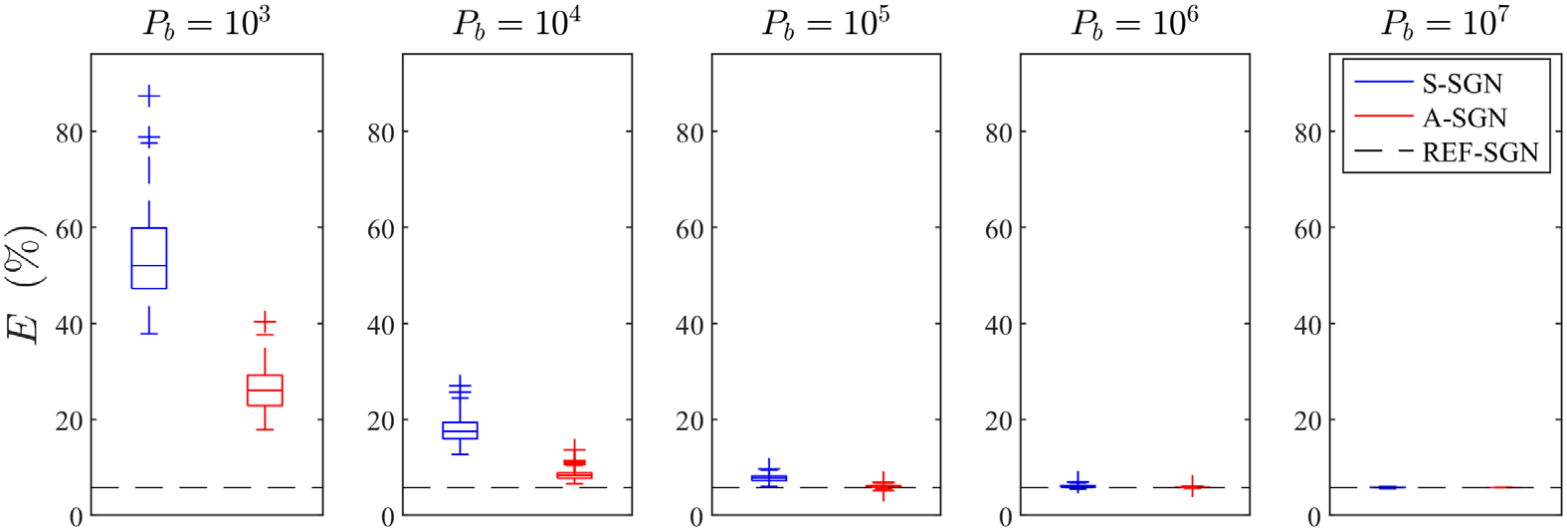
Statistics of the relative errors of the estimates with different photon budgets $P_{b}$ (images from left to right) when the domain was illuminated from all boundaries. For each photon budget: S-SGN method (blue, left), A-SGN method (red, right), and the reference reconstruction (black, horizontal dashed line). Blue and red vertical lines (whiskers) denote all the samples excluding outliers, box denotes the 25th and 75th percentile, horizontal line denotes the median, and + symbol denotes outliers.

**Fig. 10:**
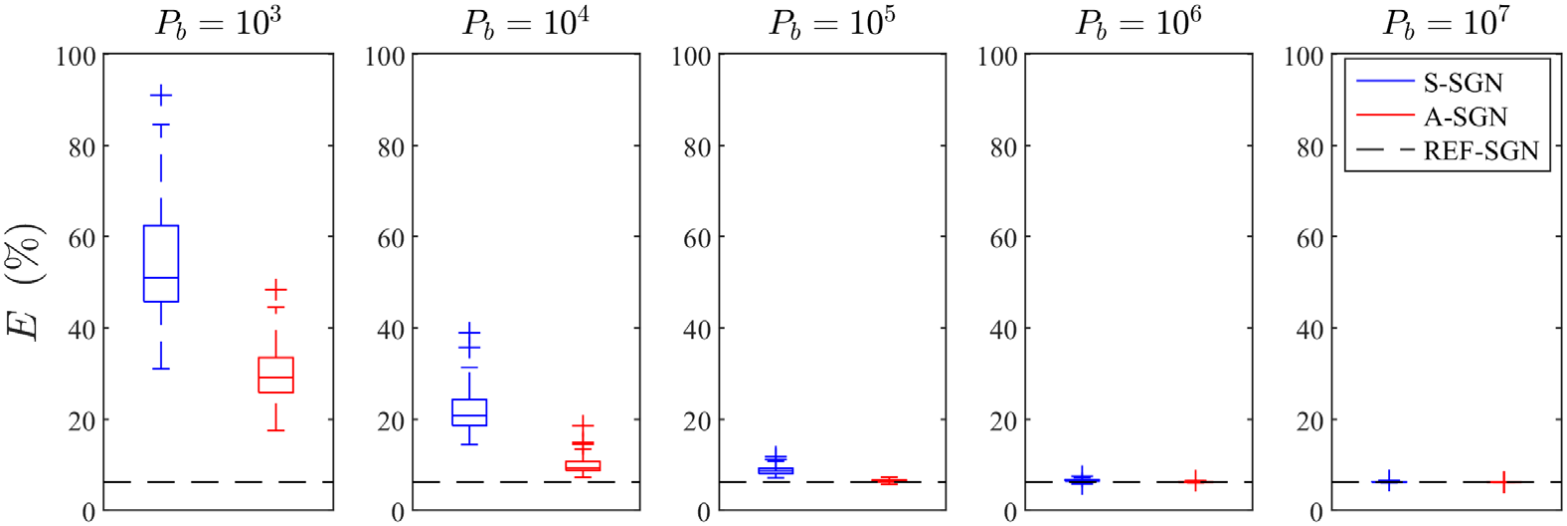
Statistics of the relative errors of the estimates with different photon budgets $P_{b}$ (images from left to right) when the domain was illuminated from top and left boundaries. For each photon budget: S-SGN method (blue, left), A-SGN method (red, right), and the reference reconstruction (black, horizontal dashed line). Blue and red vertical lines (whiskers) denote all the samples excluding outliers, box denotes the 25th and 75th percentile, horizontal line denotes the median, and + symbol denotes outliers.

The statistics of the relative errors of the reconstructions for the 100 evaluation cases are shown in Fig. 9 for the simulations where the domain was illuminated from all boundaries and in Fig. 10 for the simulations where the domain was illuminated from top and left boundaries. Further, the mean and standard deviation of the relative errors of the reconstructions are presented in Table 2. As it can be seen, with small photon budgets ($10^{3}$ and $10^{4}$), the A-SGN method is able to provide significantly lower relative errors than the S-SGN method. When the photon budget increases, the difference between these methods decreases. With photon budgets of $10^{6}$ and higher, the difference between the A-SGN and S-SGN methods is negligible, as the relative errors of both A-SGN and S-SGN are very close to the relative error of the reference estimate. When comparing the number of illuminations, it can be noticed that in general, the relative errors are smaller and absorption estimates are more accurate, when the domain has been illuminated from all boundaries when compared to illuminations only from top and bottom boundaries. Furthermore, the statistical variation of the relative errors with small photon budgets is smaller with four illuminations than with two illuminations.

**Table 2:** Mean of the relative error E(%) of the reconstructions and its standard deviation with S-SGN and A-SGN approaches, with four and two illuminations and different photon budgets $P_{b}$.

	Four illuminations		Two illuminations	
	S-SGN	A-SGN	S-SGN	A-SGN
$P_{b}=10^{3}$	54.3±10.0	25.9±4.6	57.5±12.3	29.7±6.1
$P_{b}=10^{4}$	17.9±2.9	8.5±1.1	21.2±4.1	9.8±1.7
$P_{b}=10^{5}$	7.7±0.7	6.0±0.3	8.7±1.0	6.5±0.3
$P_{b}=10^{6}$	6.0±0.3	5.8±0.1	6.5±0.3	6.2±0.1
$P_{b}=10^{7}$	5.78±0.09	5.76±0.03	6.25±0.09	6.21±0.03

### 6.3 Comparison of the Computation Times

In the third study, computation times of the approaches were compared when the domain was illuminated from all sides. To differentiate the main factors contributing to the computation time, three different times were studied: (1) “Monte Carlo time,” which is the time required to simulate photon packets both for forward problem and construction of the Jacobians during the algorithm, (2) “Gauss-Newton time,” which is computation time required to solve GN directions (S-SGN and A-SGN) and the norm test for updating the estimates (A-SGN), and (3) “total computation time,” which is the sum of Monte Carlo time and Gauss–Newton time.

The A-SGN reconstructions were computed with two different samples L=5 and L=10 used in the norm test. The S-SGN reconstructions were computed with four different number of photon packets per iteration: $10^{5}$, $10^{6}$, $10^{7}$, and $10^{8}$. The algorithms were considered converged when the relative difference between the last and three previous absorption estimates was smaller than 10% for all of the three previous estimates, similarly as in the first study.

**Fig. 11:**
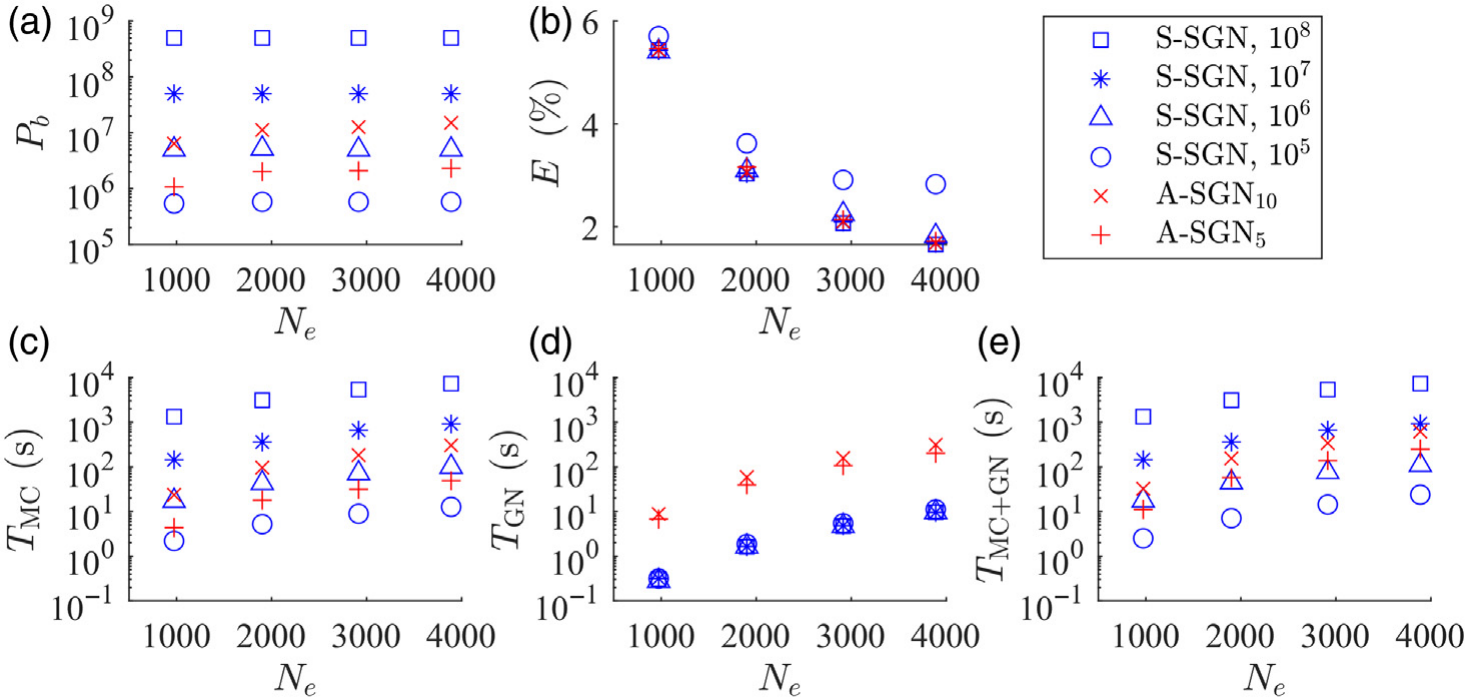
(a) Mean photon budget $P_{b}$ and (b) relative reconstruction errors E(%) with different number of discretization elements $N_{e}$. (c) Mean Monte Carlo time $T_{\mathrm{MC}}$, (d) Gauss-Newton time $T_{\mathrm{GN}}$, and (e) total computation time $T_{\mathrm{MC}+\mathrm{GN}}$ with different number of discretization elements. The S-SGN approach with a fixed number of photon packets per iteration of $10^{8}$ (□), $10^{7}$ (✳), $10^{6}$ (▵), and $10^{5}$ (○), and the A-SGN approach with 10 samples (×) and 5 samples (+).

The mean of the total number of photon packets, relative reconstruction errors and computation times in different reconstruction discretizations are shown in Fig. 11. As it can be seen, in all approaches the relative errors of the reconstructions are almost identical, except in the S-SGN approach with the lowest number of photon packets. That is, $10^{5}$ photon packets per iteration can be interpreted to be insufficient to achieve similar accuracy as the other approaches. When comparing the computation times, it can be seen that in both A-SGN and S-SGN approaches, computation times increase with an increasing number of discretization elements. Also, in both approaches, computation times increase with an increasing number of photon packets. In the A-SGN, the time required to evaluate multiple GN minimization directions is a significant part of the total computation time, and it is more time consuming with an increasing number of unknowns and data. In the S-SGN, the GN minimization direction is evaluated only once in each iteration. However, in the S-SGN, the amount of photons is fixed, and with an increasing number of discretization elements, the computation times increase significantly. This is especially evident if a large number of photon packets are simulated.

### 6.4 Three-Dimensional Simulation

Then, the A-SGN and S-SGN approached were evaluated with a 3D study. In the S-SGN approach, the number of photon packets was fixed to be $10^{8}$ per iteration. The algorithms were considered converged when the relative difference between the last and three previous absorption estimates was smaller than 10% for all of the three previous estimates.

**Fig. 12:**
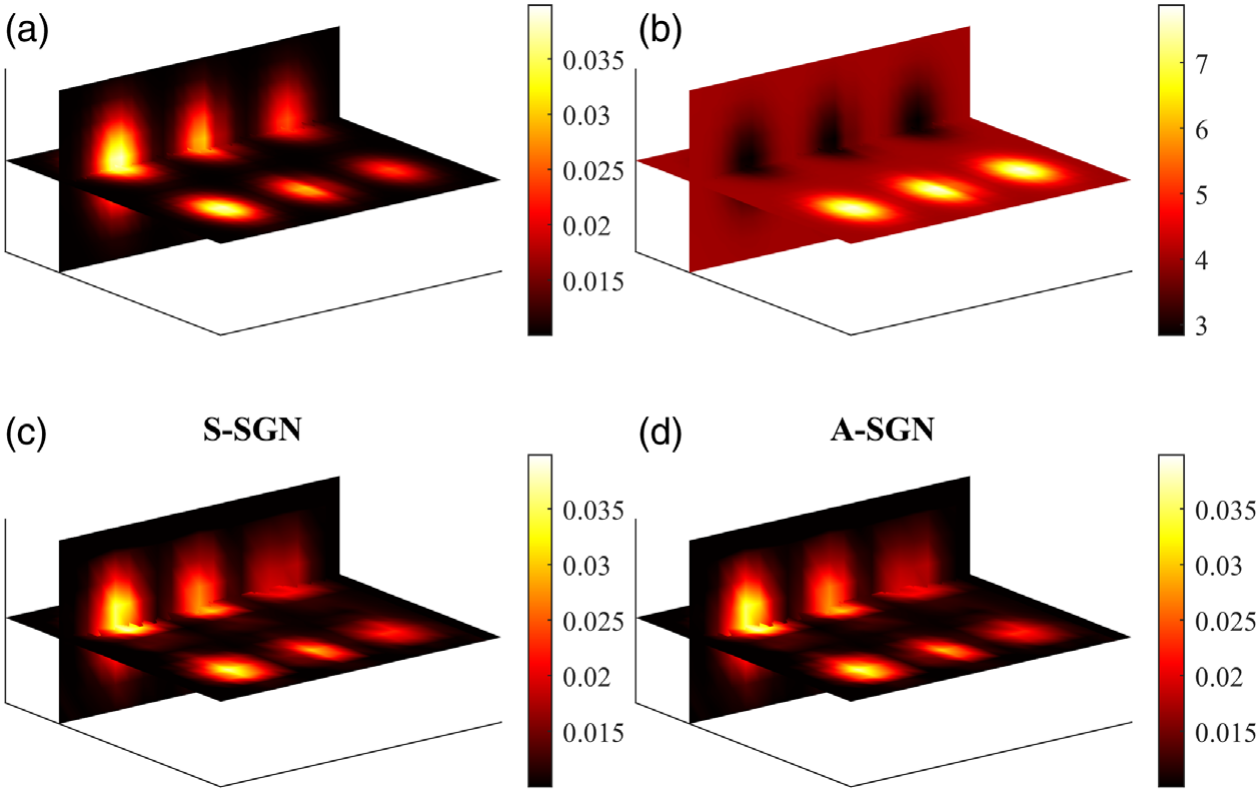
(a) Simulated absorption and (b) scattering distributions. Absorption distribution reconstructed using (c) S-SGN approach and (d) A-SGN approach.

The simulated (true) absorption and scattering distributions and the reconstructed absorption distributions obtained with the S-SGN and A-SGN method are shown in Fig. 12. Both S-SGN and A-SGN reconstructions look qualitatively identical by visual inspection. Further, the relative reconstruction error in both approaches was 10%. The total number of photon packets utilized in the S-SGN approach was $5\times 10^{8}$ and in the A-SGN approach $1.1\times 10^{7}$.

## 7 Discussion and Conclusions

In this work, an A-SGN method was proposed for the solution of the image reconstruction problem of quantitative photoacoustic tomography. In the approach, the QPAT image reconstruction problem was formulated as a minimization problem. This problem was solved with a SGN method with a Monte Carlo light transport method as the forward model for light propagation. An approach for adaptively determining the number of photon packets on each iteration was proposed. The approach was based on a norm test where the expected squared relative error of minimization direction was controlled. Similar stochastic optimization problems can be found in a high-dimensional machine learning setting, where the size of the training data set is often so large, that computing for example gradient of the cost function for the full training data set is computationally infeasible.^55^

The presented approach was studied with numerical simulations. Compared to a S-SGN method, where the number of photon packets was fixed, the adaptive method provided reconstructions with similar relative errors with significantly lower photon budgets. It was also shown that the adaptive approach can provide similar quality reconstructions as a reference approach with a very large number of photon packets. When comparing computation times in different discretizations, it was seen that the adaptive approach required less time to simulate the forward solution and to construct the Jacobians than the conventional approach. On the other hand, it required multiple evaluations of the GN search direction on each iteration and in that regard, it was slower than the conventional approach. Still, the adaptive approach provided significant savings in computation times compared to a simple SGN approach with a large number of photon packets. It should also be remembered that knowing an optimal fixed number of photon packets for an algorithm may be difficult beforehand. In the adaptive approach, the number of photon packets is adjusted automatically, and a convergence to desired criterion can be achieved.

The adaptive approach necessitates choosing multiple parameters that affect the effectiveness of the approach: accepted error in the minimization direction, how often the norm test is evaluated, and the number of samples utilized in the norm test. Overall, many factors such as the geometry of the imaged domain, discretization, and optical parameters affect on the minimization problem. In this work, the parameters of the adaptive algorithm were chosen by repeated simulations with different parameter values. However, more research is required for determining them in different imaging scenarios and further utilization of the methodology.

In this work, the scattering coefficient was assumed to be known. In practice, this is not necessarily a realistic assumption. The approach presented in this work could be applied to estimation of both absorption and scattering coefficients where the evaluation of the Jacobians requires utilization of an approximation such as the perturbation Monte Carlo method.^39^ Implementation and effect of this approximation on stochastic Monte Carlo implementations remain as a future research direction. Furthermore, the discretizations utilized in this work were relatively coarse. In addition, only the optical part of the QPAT problem was studied, without considering the acoustic reconstruction and its possible effects on the data. Accuracy and computational efficacy of Monte Carlo-based inversion methods in more realistic simulations require thus further work.

In conclusion, utilizing the SGN method with a Monte Carlo light transport model in QPAT can provide accurate reconstructions. Furthermore, adaptively determining the number of photon packets during iterations can be utilized to minimize the simulation of unnecessary photon packets in the image reconstruction, thus reducing the computational cost of the inverse Monte Carlo method.
